# Supplementary material for: Asynchronous magnetic resonance elastography: Shear wave speed reconstruction using noise correlation of incoherent waves
Source: Magn Reson Med. 2022 Oct 27;89(3):990–1001. doi: 10.1002/mrm.29502 (PMC9792433; doi:10.1002/mrm.29502)
Supplement: Supplementary file 1 — DATA S1 MATLAB code used to generate the numerical simulations of Figure 1 [file MRM-89-990-s002.zip › k-Wave/helpfiles/gradientFD.html]

gradientFD :: Functions (k-Wave)


# gradientFD

Calculate the gradient using a finite-difference method.

## Syntax

```
fx = gradientFD(f, dx)
fx = gradientFD(f, dx, [], deriv_order)
fx = gradientFD(f, dx, [], deriv_order, accuracy_order)
fn = gradientFD(f, dn, dim)
fn = gradientFD(f, dn, dim, deriv_order, accuracy_order)
[fx, fy] = gradientFD(f, dn)
[fx, fy] = gradientFD(f, dn, [], deriv_order, accuracy_order)
[fx, fy, fz, ...] = gradientFD(f, dn)
[fx, fy, fz, ...] = gradientFD(f, dn, [], deriv_order, accuracy_order)
```

## Description

`gradientFD` calculates the gradient of an n-dimensional input matrix using the finite-difference method. For one-dimensional inputs, the gradient is always computed along the non-singleton dimension. For higher dimensional inputs, the gradient for singleton dimensions is returned as 0. For elements in the centre of the grid, the gradient is computed using centered finite-differences. For elements on the edge of the grid, the gradient is computed using forward or backward finite-differences. The order of accuracy of the finite-difference approximation is controlled by `accuracy_order` (default = 2). The calculations are done using sparse multiplication, so the input matrix is always cast to double precision.

A 1D example:

```
% compute gradient of a 2 period sinusoid
x = pi/20:pi/20:4*pi;
y = sin(x);
dydx_4th_ord = gradientFD(y, pi/20, [], 1, 4);

% plot gradient and error compared to analytical solution
subplot(2, 1, 1), plot(x, cos(x), 'k-', x, dydx_4th_ord, 'bx');
axis tight;
title('dy/dx');
subplot(2, 1, 2), plot(x, cos(x) - dydx_4th_ord, 'k-');
axis tight;
title('Relative Error');
```

## Inputs

|  |  |
| --- | --- |
| `f` | matrix or vector to find the gradient of |
| `dn` | array of values for the grid point spacing in each dimension. If a value for `dim` is given, `dn` is the spacing in dimension `dim`. |

## Optional Inputs

|  |  |
| --- | --- |
| `dim` | optional input to specify a single dimension over which to compute the gradient for n-dimension input functions |
| `deriv_order` | order of the derivative to compute, e.g., use 1 to compute df/dx, 2 to compute df^2/dx^2, etc. (default = 1) |
| `accuracy_order` | order of accuracy for the finite difference coefficients. Because centered differences are used, this must be set to an integer multiple of 2 (default = 2) |

## Outputs

|  |  |
| --- | --- |
| `fx, fy, ...` | gradient in the each dimension, where x corresponds to dim = 1, y corresponds to dim = 2 etc. |

## See Also

`getFDMatrix`, `gradient`, `gradientSpect`
